# Supplementary material for: A UMLS-based spell checker for natural language processing in vaccine safety
Source: BMC Med Inform Decis Mak. 2007 Feb 12;7:3. doi: 10.1186/1472-6947-7-3 (PMC1805499; doi:10.1186/1472-6947-7-3)
Supplement: Additional file 13 — RAP application directory. Sets up directory for RAP (RDF application for PHP) source code [file 1472-6947-7-3-S13.gz › rap/api/util/adodb/tests/testcache.php]

php
/\*
V4.52 10 Aug 2004 (c) 2000-2004 John Lim (jlim@natsoft.com.my). All rights reserved.
Released under both BSD license and Lesser GPL library license.
Whenever there is any discrepancy between the two licenses,
the BSD license will take precedence.
Set tabs to 4 for best viewing.
Latest version is available at http://adodb.sourceforge.net
\*/
$ADODB\_CACHE\_DIR = dirname(tempnam('/tmp',''));
include("../adodb.inc.php");
if (isset($access)) {
$db=ADONewConnection('access');
$db-PConnect('nwind');
} else {
$db = ADONewConnection('mysql');
$db->PConnect('mangrove','root','','xphplens');
}
if (isset($cache)) $rs = $db->CacheExecute(120,'select \* from products');
else $rs = $db->Execute('select \* from products');
$arr = $rs->GetArray();
print sizeof($arr);
?>
